# Drug Reaction With Eosinophilia and Systemic Symptoms to Vancomycin-Laden Cement Space: A Case Report

**DOI:** 10.7759/cureus.76860

**Published:** 2025-01-03

**Authors:** Sai Chalasani, Harshitha Mannam, Ahmed K Alomari, Sahand Rahnama-Moghadam

**Affiliations:** 1 Dermatology, Indiana University School of Medicine, Indianapolis, USA; 2 Dermatology/Pathology, Indiana University School of Medicine, Indianapolis, USA

**Keywords:** antibiotic-loaded cement spacer, dress syndrome, drug-related side effect, vancomycin-induced dress syndrome, vancomycin-loaded bone cement

## Abstract

Drug reaction with eosinophilia and systemic symptoms (DRESS) is a severe cutaneous adverse drug reaction mediated by a complex immune response. Vancomycin is a known cause of DRESS, and cases are often attributed to intravenous exposure. Vancomycin-laden bone cements deliver high concentrations of the drug locally with low to undetectable systemic levels. Despite trace systemic concentrations, cement spacers have been reported to cause systemic reactions ranging from organ failure to diffuse cutaneous eruptions.

A patient receiving intravenous (IV) and local vancomycin, via bone cement, experienced symptom resolution only after the vancomycin-eluting bone cement was removed, which was done after the IV vancomycin had been stopped. This suggests that the vancomycin eluted from the local bone cement may be sufficient to maintain the immune response mediating DRESS syndrome. In patients who experience persistent symptoms despite discontinuing systemic drug exposure, clinicians should consider eliminating all sources of the causative drug.

## Introduction

Drug reaction with eosinophilia and systemic symptoms (DRESS) is an uncommon severe cutaneous adverse drug reaction that can present with severe skin eruption and systemic findings including fever, lymphadenopathy, hematologic abnormalities, and internal organ involvement. 

The pathogenesis of DRESS is thought to be an interaction between three processes including a genetic component, the activation of the immune system related to a causative drug, and perhaps a triggering factor, postulated to be viral reactivation [1]. There is activation of virus-specific and nonspecific T cells that target the drug and mediate tissue destruction. Symptom onset is often delayed to 2-8 weeks after drug exposure [2]. The rash is typically morbilliform and often does concern for a drug rash, the internal organ involvement being a distinguishing feature of DRESS syndrome. The long latency period of 2-8 weeks may often cloud the diagnosis as this allows more opportunity for multiple drugs to have been added at this time. Antibiotics are common causes of DRESS syndrome.

Antibiotic-laden cement spacers disperse a high concentration of drugs locally with low to undetectable systemic levels to treat joint infections with high success rates [3]. While most studies have found that antibiotic-laden cement spacers are safer than systemic antibiotic exposure, there are reports of complications such as liver dysfunction and acute renal failure from cement spacers [4-6]. A cement spacer is typically used in joint replacement procedures to reduce the risk of infection. Here we report a case of vancomycin-laden cement spacer contributing to DRESS syndrome. In this case, despite an appropriate dose of systemic steroids, DRESS syndrome was only controlled when the vancomycin-laden cement spacer was removed surgically to remove any trace amount of vancomycin that was driving the syndrome.

## Case presentation

A 46-year-old previously healthy woman was admitted to the hospital from a clinic for fever, malaise, a diffuse pruritic eruption, and anasarca during the third week of a planned six-week vancomycin antibiotic course to treat culture-proven Propionibacterium acnes (P. acnes) osteomyelitis. To treat the osteomyelitis, a total shoulder arthroplasty was performed to remove the infected bone, then a temporary orthopedic bone cement with vancomycin (2g) and tobramycin (2.4g) was placed in the joint to clear any biofilm, and IV vancomycin was initiated. 

After two weeks, the patient began to experience the insidious onset of pruritus and red edematous plaques starting around the incision site of the arthroplasty and cement placement. She developed a diffuse red morbilliform eruption, anasarca, and severe pruritus that was uncontrolled despite oral diphenhydramine 25mg every 6 to 8 hours. Having never experienced this reaction before, nor having been warned about this possibility, she reported to the infectious disease clinic complaining of rash, fever, dysphagia, dyspnea, and nausea. IV vancomycin was discontinued and oral prednisone at 30mg once daily was initiated. However, within 24 hours her symptoms worsened and subsequently, she was admitted for the fever, rash, and constitutional symptoms. 

**Figure 1:**
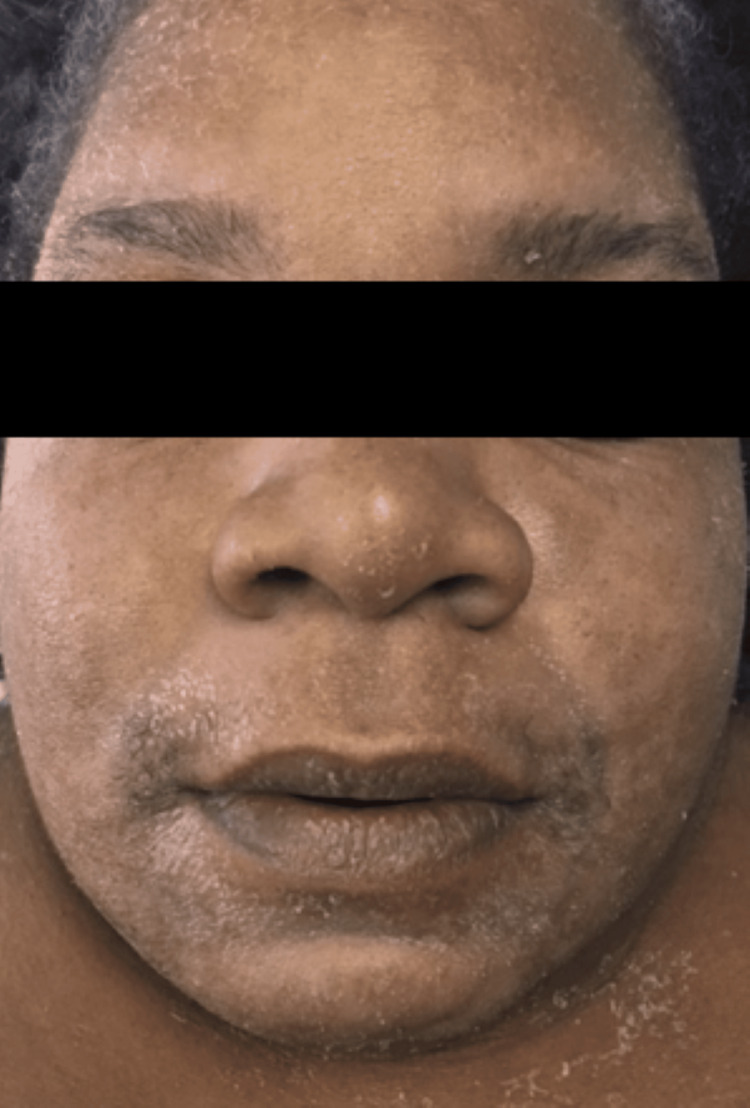
Facial edema with morbilliform eruption and fine desquamation

**Figure 2:**
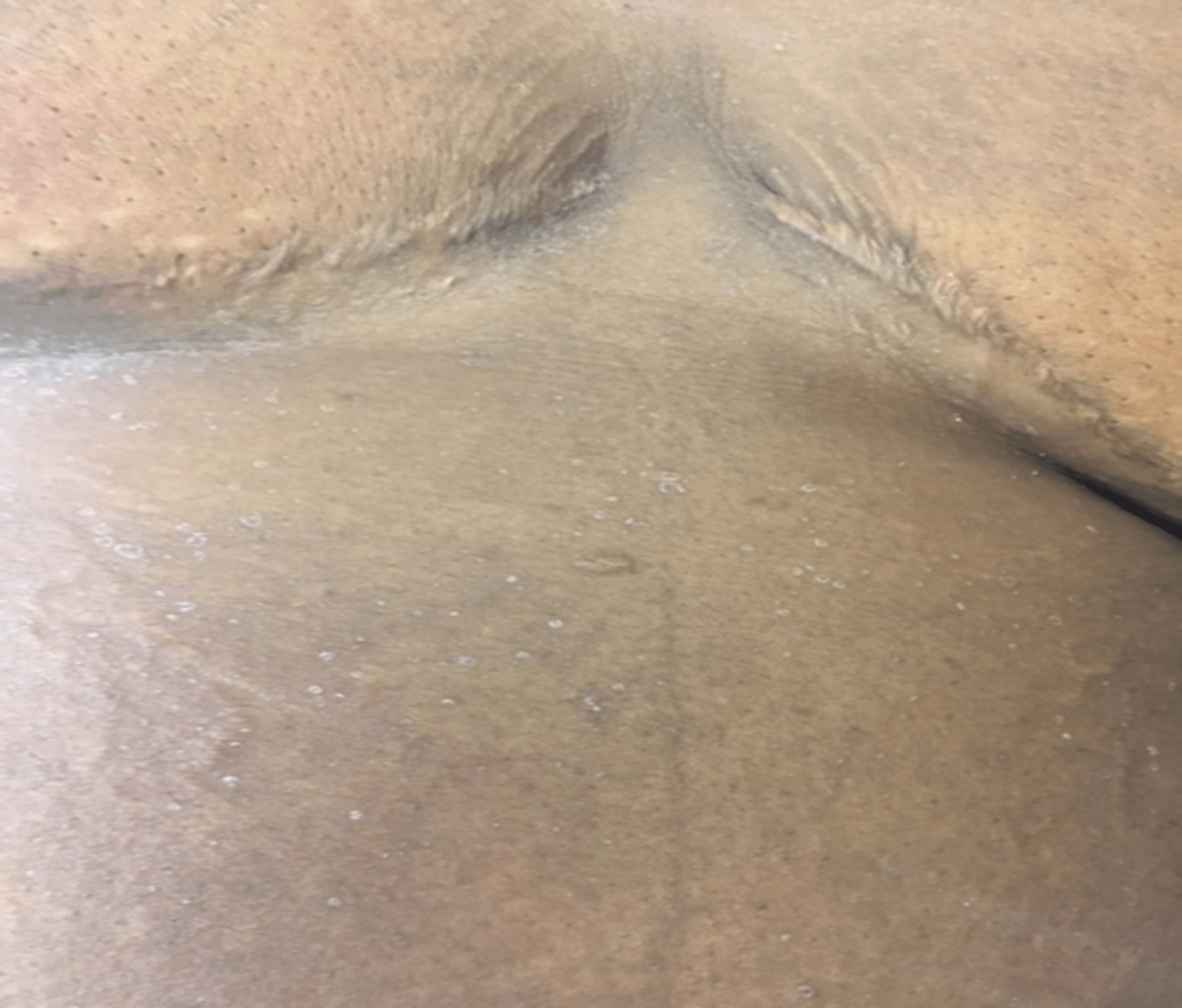
Truncal morbilliform eruption with follicular accentuation and fine desquamation

**Figure 3:**
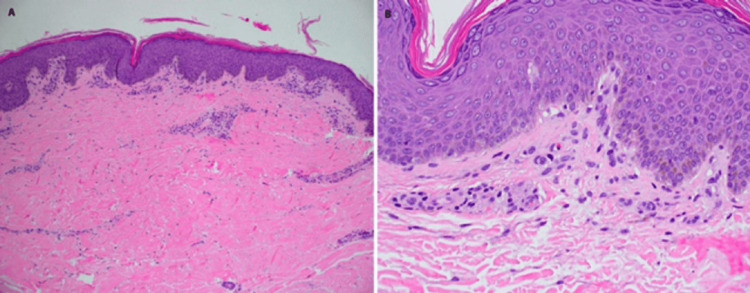
Skin biopsy showing superficial perivascular infiltrate with occasional eosinophils and minimal spongiosis

On exam, she was found to be febrile to a temperature of 38 degrees Celsius (100.8 degrees F), tachycardic, and slightly hypotensive at 96/73. She was ill-appearing, debilitated, and anasarcic with obvious facial edema (see Figures 1, 2). She had a generalized, faintly erythematous morbilliform eruption to the trunk and proximal extremities with perifollicular accentuation and multifocal, mobile, tender lymphadenopathy in the bilateral anterior cervical, inguinal, and axillary regions all measuring approximately 1 centimeter. Her labs revealed an elevated white blood cell count (WBC) at 15.1 x 103/ul (reference range: 3.6 to 10.6 k/mL), unremarkable neutrophil count, and elevated eosinophil count at 1500 cells/uL (reference range: 0 to 0.3 k/mL). Her baseline eosinophils had been normal before initiation of IV vancomycin and placement of the antibiotic spacer. Her serum creatinine was also elevated at 1.2mg/dL (reference range: 0.8 to 1.4 mg/dL), while her blood urea nitrogen was normal and unchanged. Hepatic enzyme levels were normal. Urinalysis showed an increase in the WBC with no detected bacteria. Blood and urine cultures did not reveal infection. A clinical diagnosis of DRESS syndrome was made, supported by a biopsy of this eruption showing a superficial perivascular and interstitial infiltrate with dermal eosinophils and minimal epidermal spongiosis consistent with DRESS syndrome. The lack of significant spongiosis militated against a diagnosis of a contact or atopic dermatitis flare (see Figure 3). 

The dermatology service discussed the case with the primary team and the orthopedic surgery service. While there was agreement on starting higher doses of prednisone at 0.5mg/kg/day (60mg daily), there was clinical equipoise regarding the removal of antibiotic spacer as there was scant literature available for guidance and the supposition that the vancomycin exposure should be mostly limited to the joint space. After five days of the higher dose of prednisone therapy, the patient had stabilization in her pruritus, eosinophil count, and creatinine level and the patient was discharged with close dermatology follow-up. 

**Figure 4:**
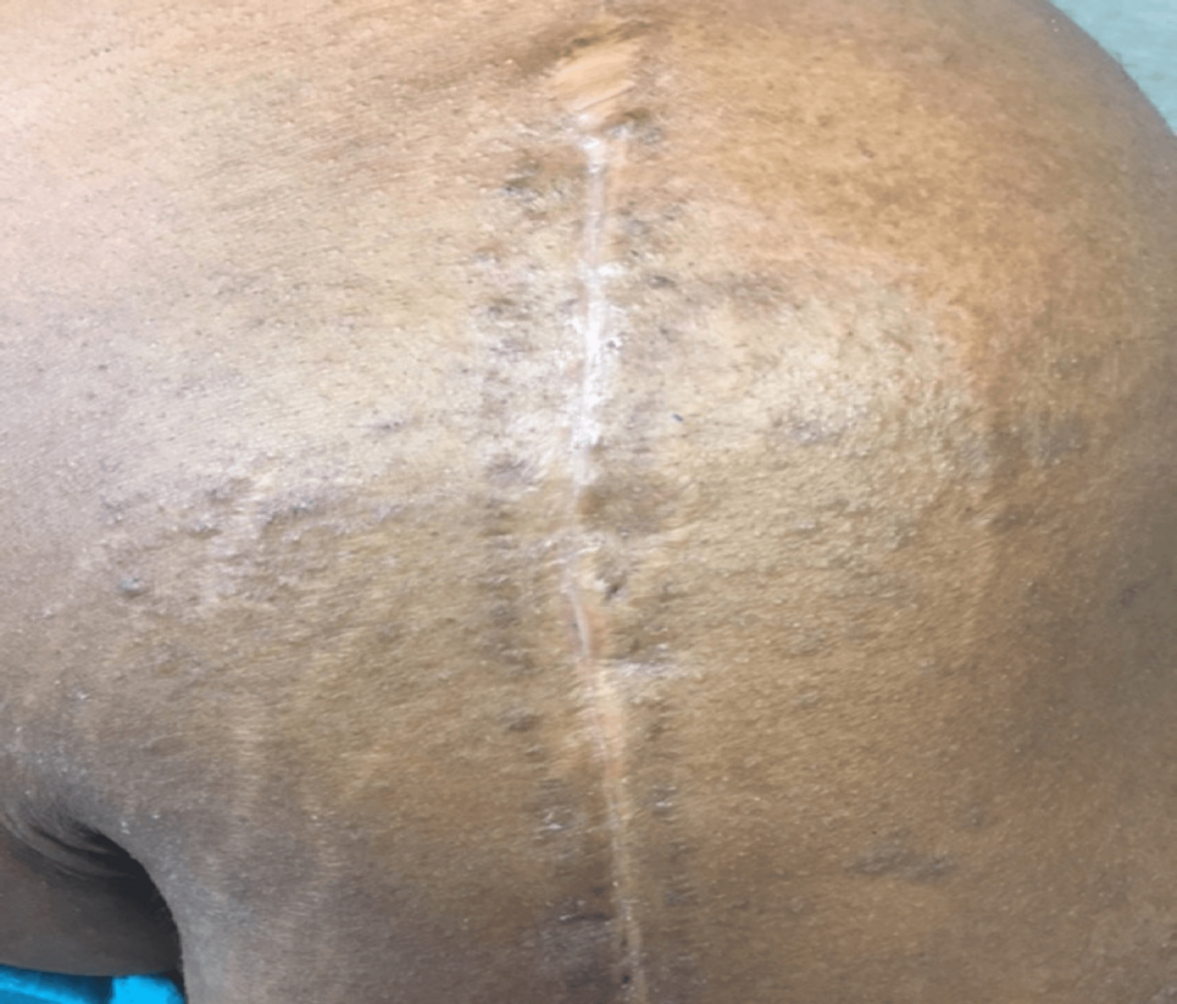
Left shoulder arthroplasty site well-healed with continous surrounding lichenified papules

However, less than 24 hours after discharge from the hospital, she had a recrudescence of her symptoms while on prednisone 60mg daily. On presentation to the ED, she had a high fever of 39.1 C (102.4 F), severe headaches, photophobia, dyspnea, pruritus, worsened eruption, and recurrence of facial edema. At this presentation, her white count was normal, and her absolute eosinophil count was only 800. Her creatinine was normal. However, she had an elevation in her AST at 105 U/L (reference range: 13 to 39 units/L), ALT at 68 U/L (reference range: 7 to 52 units/L), and alkaline phosphatase at 168 U/L (reference range: 25 to 125 units/L). After ruling out infection, her condition was felt to be consistent with recrudescent DRESS syndrome and it was decided to have the antibiotic spacer removed while continuing the same dose of prednisone 60mg daily. The next day, after surgical removal of the spacer, the patient noted improved pruritus and energy levels. Her pruritus, energy level, and skin rash improved steadily while her liver enzymes and eosinophil counts improved in a continuous fashion. Her prednisone requirements were able to be steadily tapered to zero over the period of six weeks while her systemic symptoms resolved. After the spacer was removed, her symptoms were never undulant and rather improved continuously. On follow-up, she did note her rash and pruritus were nearly resolved but continued slightly at the surgical site where the spacer was placed (see Figure 4). This was resolved with topical triamcinolone. 

## Discussion

DRESS syndrome can be difficult to identify due to the range of cutaneous and systemic findings. The morbilliform eruption, constitutional symptoms, 2-8-week latency period with causative medication of vancomycin, elevated eosinophil count, lymphadenopathy, creatinine elevation suggestive of acute interstitial nephritis, and characteristic biopsy indicate DRESS. While our patient’s recrudescent symptoms and ultimate improvement could be due to an undulant clinical course, the fact that the spacer elutes vancomycin and the condition improved only with the removal of the spacer and the improvement itself was steady and the course no longer undulant after removal is suggestive of the point that all potential sources of the causative agent should be removed. 

Medium-dose antibiotic spacers often have undetectable systemic levels of antibiotics [3], but there have been three reports of diffuse cutaneous manifestations to local vancomycin-laden bone cement [7-9]. Symptoms resolved after removal of cement spacer and suggests that local vancomycin can cause diffuse cutaneous reactions despite undetectable to low systemic levels and supports the plausibility of the antibiotic spacer being the culprit in this case as well.

Activated T cells are thought to mediate systemic symptoms [1], but the amount of drug necessary to sustain the immune reaction in DRESS syndrome has not been described. In two previous case reports of DRESS, patients with both IV and local vancomycin exposure experienced symptom resolution after discontinuing IV vancomycin [10]. Other factors such as genetics and drug metabolism contribute to patient predisposition to developing DRESS. As a hypothesis, it is possible that trace levels of the culprit drug are sufficient to perpetuate an immune response in a sensitized patient. This patient’s condition improved rapidly and was only controlled once the antibiotic spacer was removed which suggests a likely role for the spacer in maintenance of her disease and may pertain to other patients with persistent symptoms with antibiotic spacers. 

## Conclusions

Vancomycin is a recognized cause of DRESS syndrome, and mostly systemic exposure was identified as the culprit. Our patient’s symptoms improved steadily after the cement spacer was removed and only steadily improved once removed, suggesting that the spacer contributed to the systemic reaction. The low systemic concentration eluted from the spacer was likely enough to continue the immune response responsible for DRESS syndrome in sensitized patients. It is important to be cognizant of antibiotic-laden cement spacers as a potential factor in disease maintenance of recalcitrant DRESS and to emphasize that once a causative agent is identified, all sources of that drug may need to be removed.

## References

[1] Choudhary S, McLeod M, Torchia D, Romanelli P (2013). Drug reaction with eosinophilia and systemic symptoms (DRESS) syndrome. J Clin Aesthet Dermatol.

[2] Cacoub P, Musette P, Descamps V, Meyer O, Speirs C, Finzi L, Roujeau JC (2011). The DRESS syndrome: a literature review. Am J Med.

[3] Luu A, Syed F, Raman G (2013). Two-stage arthroplasty for prosthetic joint infection: a systematic review of acute kidney injury, systemic toxicity and infection control. J Arthroplasty.

[4] Kalil GZ, Ernst EJ, Johnson SJ, Johannsson B, Polgreen PM, Bertolatus JA, Clark CR (2012). Systemic exposure to aminoglycosides following knee and hip arthroplasty with aminoglycoside-loaded bone cement implants. Ann Pharmacother.

[5] Koo KH, Yang JW, Cho SH (2001). Impregnation of vancomycin, gentamicin, and cefotaxime in a cement spacer for two-stage cementless reconstruction in infected total hip arthroplasty. J Arthroplasty.

[6] Patrick BN, Rivey MP, Allington DR (2006). Acute renal failure associated with vancomycin- and tobramycin-laden cement in total hip arthroplasty. Ann Pharmacother.

[7] Xu S, Ponce BA, Pavlidakey PG, Brabston EW 3rd (2016). Drug eruption secondary to vancomycin-laden spacer in the shoulder: a case report. J Shoulder Elbow Surg.

[8] Williams B, Hanson A, Sha B (2014). Diffuse desquamating rash following exposure to vancomycin-impregnated bone cement. Ann Pharmacother.

[9] Riemenschneider K, Diiorio DA, Zic JA (2018). Drug-induced linear IgA bullous dermatosis in a patient with a vancomycin-impregnated cement spacer. Cutis.

[10] Güner MD, Tuncbilek S, Akan B, Caliskan-Kartal A (2015). Two cases with HSS/DRESS syndrome developing after prosthetic joint surgery: does vancomycin-laden bone cement play a role in this syndrome?. BMJ Case Rep.

